# Reconstruction and analysis of nutrient-induced phosphorylation networks in *Arabidopsis thaliana*

**DOI:** 10.3389/fpls.2013.00540

**Published:** 2013-12-24

**Authors:** Guangyou Duan, Dirk Walther, Waltraud X. Schulze

**Affiliations:** ^1^Max Planck Institute of Molecular Plant PhysiologyPotsdam, Germany; ^2^Department of Plant Systems Biology, Universität HohenheimStuttgart, Germany

**Keywords:** phosphorylation, protein–protein interaction, signaling transduction, network inference, correlation, *Arabidopsis thaliana*

## Abstract

Elucidating the dynamics of molecular processes in living organisms in response to external perturbations is a central goal in modern systems biology. We investigated the dynamics of protein phosphorylation events in *Arabidopsis thaliana* exposed to changing nutrient conditions. Phosphopeptide expression levels were detected at five consecutive time points over a time interval of 30 min after nutrient resupply following prior starvation. The three tested inorganic, ionic nutrients NH^+^_4_, NO^−^_3_, PO^3−^_4_ elicited similar phosphosignaling responses that were distinguishable from those invoked by the sugars mannitol, sucrose. When embedded in the protein–protein interaction network of *Arabidopsis thaliana*, phosphoproteins were found to exhibit a higher degree compared to average proteins. Based on the time-series data, we reconstructed a network of regulatory interactions mediated by phosphorylation. The performance of different network inference methods was evaluated by the observed likelihood of physical interactions within and across different subcellular compartments and based on gene ontology semantic similarity. The dynamic phosphorylation network was then reconstructed using a Pearson correlation method with added directionality based on partial variance differences. The topology of the inferred integrated network corresponds to an information dissemination architecture, in which the phosphorylation signal is passed on to an increasing number of phosphoproteins stratified into an initiation, processing, and effector layer. Specific phosphorylation peptide motifs associated with the distinct layers were identified indicating the action of layer-specific kinases. Despite the limited temporal resolution, combined with information on subcellular location, the available time-series data proved useful for reconstructing the dynamics of the molecular signaling cascade in response to nutrient stress conditions in the plant *Arabidopsis thaliana*.

## Introduction

Intra-cellular communication and information processing is performed by complex, dynamic, and context-specific molecular signaling networks (Terfve and Saez-Rodriguez, 2012). Protein phosphorylation is the best known post-translational modification involved in molecular signaling and plays a significant role in a wide range of cellular processes in all organisms (Cohen, 2000; Macek et al., 2008; Schulze, 2010). Enabled by the development of novel sensitive experimental techniques to detect phosphorylation sites in proteins (Larsen et al., 2005; Sugiyama et al., 2007), the principle of phosphorylation-mediated activation and inactivation of proteins and the modulation of molecular interactions has been studied intensively in a broad range of organisms (Pawson, 1997; Pawson and Nash, 2003; Pawson and Taylor, 2009). Time course studies of phosphorylation events are of particularly high value in the context of signaling as they allow to reveal the dynamics and the cause-effect relationships between all molecular components involved in the signal transduction process (Blagoev et al., 2004; Olsen et al., 2006; Niittylä et al., 2007; Engelsberger and Schulze, 2012).

Besides advances on the instrumentation side, computational methods have been devised to interrogate the resulting data sets. Both descriptive as well as predictive computational methods have been developed [For review, see Janes and Yaffe (2006)]. In the category of *descriptive* approaches, in particular the methods clustering (Olsen et al., 2006; Huang et al., 2007; Engelsberger and Schulze, 2012), principal component analysis (PCA), singular value decomposition (SVD), data mapping onto protein interaction networks (PIN), and protein signaling networks (PSN) (Krüger et al., 2008; Jørgensen et al., 2009) have been applied with the aim to reduce the complex behavior of a molecular system to the core components, thereby “describing” it in the most meaningful and parsimonious way. The primary goal of descriptive approaches is to identify common as well as distinguishing properties of the system under study exposed to different conditions or at different time points. By contrast, *predictive* approaches allow predicting the system's response given a set of conditions and data for appropriate independent predictor variables (Terfve and Saez-Rodriguez, 2012). Predictive approaches employ input/output regression based methods (such as partial least squares regression (Gaudet et al., 2005; Janes et al., 2005), multiple linear regression (Ekins et al., 2008; Alexopoulos et al., 2010) as well methods that aim to infer (in a sense “predict”) the web of interactions between all components based on the available data using network inference methods [such as correlation based inference methods (Ciaccio et al., 2010), modular response analysis (Kholodenko, 2006; Santos et al., 2007), multiple input multiple output models (Nelander et al., 2008), Bayesian network inference (Sachs et al., 2005; Ciaccio et al., 2010)] or reaction-based models such as ordinary and partial differential equations (Aldridge et al., 2006; Birtwistle and Kholodenko, 2009; Chen et al., 2009), rule-based models (Borisov et al., 2005; Conzelmann et al., 2006; Hlavacek et al., 2006; Danos et al., 2007; Faeder et al., 2009; Feret et al., 2009; Sneddon et al., 2011), and logic-based models (Gat-Viks and Shamir, 2007; Watterson et al., 2008; Saez-Rodriguez et al., 2009; Morris et al., 2010, 2011; Schlatter et al., 2011).

The field of network reconstruction gained particularly strong traction in the context of gene expression regulation, where reverse engineering models have been designed to infer gene regulatory networks from gene expression data (Bansal et al., 2007; Hecker et al., 2009). More recently, efforts were undertaken to use the generated large-scale experimental phosphorylation site data that are now available in public databases to reconstruct kinase-specific phosphorylation interactions, many of which also make use of “known” protein–protein interactions (Linding et al., 2007; Xue et al., 2008; Song et al., 2012; Newman et al., 2013).

In the plant research field, phosphoproteomic experiments interrogating the dynamics of phosphorylation events by capturing more than two time points are still very scarce (Niittylä et al., 2007; Chen et al., 2010; Engelsberger and Schulze, 2012). By contrast, pairwise comparisons of two conditions have been carried out rather frequently (Benschop et al., 2007; Li et al., 2009; Reiland et al., 2009; Kline et al., 2010).

As sessile organisms, plants have evolved sensitive mechanisms in their plasma membrane to detect and respond to rapid changes in external nutrient conditions [e.g., reviewed for responses to nitrate in Wang et al. (2012b)]. As still very little is known about post-translational regulation of nutrient-induced signaling processes, these phosphoproteomic experiments complement the existing knowledge gained from the analysis of nutrient-induced transcript changes (Scheible et al., 1997; Morcuende et al., 2007; Osuna et al., 2007; Krouk et al., 2010).

In this study, phosphoproteomics data obtained from starvation-resupply experiments involving several different nutrient conditions (nitrate, phosphate, ammonium, and the sugars mannitol and sucrose) and sampling at five consecutive time points (Niittylä et al., 2007; Engelsberger and Schulze, 2012) were analyzed. In the published experiments, mannitol has served as osmotic control and was also included here to define osmotic responses associated with nutrient changes. Based on the time-resolved phosphoproteomic data set, we conducted a systematic computational analysis of the dynamics of the observed in nutrient-induced phosphorylation events by applying descriptive approaches including clustering, data mapping onto PINs as well as predictive approaches resulting in dynamic phosphorylation network reconstructions. The objectives of this study were (a) to identify commonalities as well as characteristic differences of the phosphosignaling in response to different nutrient responses, and (b), to exploit the available time-series dataset to test various network reconstruction methods and performance metrics for their suitability to generate plausible networks even if only short time series data serve as input.

Our results demonstrate that current proteomics technologies allow monitoring of dynamic phosphorylation cascades at sufficient resolution and precision amenable for an application of computational network inference methods. The identified networks are characterized by distinct topological layers involved in signal initiation, processing, and an effector level. With time progression, we observed an increasingly broadened recruitment of phosphoproteins characteristic of an information dissemination flow in response to nutrient stimulation. Specific phosphorylation peptide motifs associated with them were identified indicating the action of layer-specific kinases.

## Results

The dataset used in this study consists of phosphopeptide identifications from starvation-resupply time course experiments (Niittylä et al., 2007; Engelsberger and Schulze, 2012) and is available from the PhosPhAt database (Durek et al., 2010). Label-free ion intensity quantitation including retention time alignment was carried out to obtain quantitative dynamic information across five time points of phosphopeptide abundance upon resupply to starved seedlings with five nutrients/organic substances (NH^+^_4_, NO^−^_3_, PO^3−^_4_, sucrose, and mannitol). As most network reconstruction methods rely on the data to be complete, only datasets with quantitative information for all five time points were retained for further analysis (Table 1). In total, 546 different peptide sequences were used in the analysis.

**Table 1:** **Number of phosphorylated peptides and proteins in each nutrient/organic solute starvation-resupply treatment with complete quantitative information across all five time points**.

	**Mannitol**	**NH_4_**	**NO_3_**	**PO_4_**	**Sucrose**
Peptides	180	76	86	144	60
Proteins	157	70	75	129	53

### Characteristics of the condition-specific phosphoprotein data sets

#### Phosphoprotein-set comparison across different nutrient conditions

**Figure 1:**
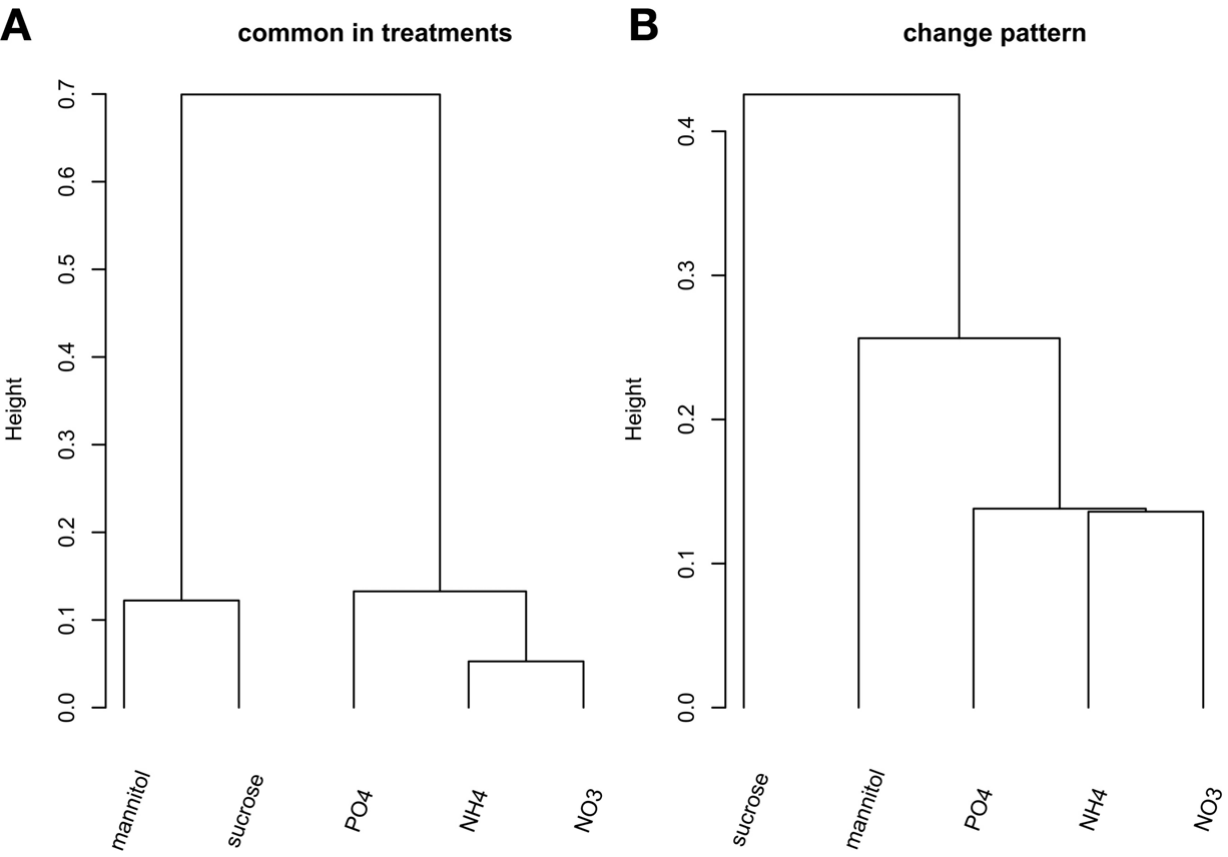
**Hierarchical clustering (“complete” linkage method) of the five nutrient/organic solute treatments. (A)** Clustering based on the inverse of the ratio between actual number of common proteins and the associated expected number based on PhosPhAt. **(B)** Clustering of the treatments based on the change pattern frequency.

We first aimed to characterize the phosphorylation events following different nutrient and osmotic challenges based on the presence and absence of phosphorylated proteins. Thus, initially we ignored the dynamics of any changes of the phosphorylation state of individual phosphorylation sites. Some nutrient/osmotic treatments exhibited a greater than expected overlap of their respective phosphoprotein set indicative of similar signaling response processes (Table 2, File S5). For example, while only one protein would be expected to be observed in common between NO^−^_3_ and NH^+^_4_ treatment when drawn randomly, 19 phosphoproteins were actually detected under both conditions. By contrast, other treatments are characterized by less similar phosphoprotein sets pointing to distinct phosphoprotein-signaling events, such as under the osmotic mannitol treatment and NH^+^_4_ nutrient treatment conditions. Here, the number of commonly observed phosphoproteins is close to random. The overlap of the commonly identified proteins for all nutrient conditions is visualized by hierarchically clustering the inverse of the ratio between actually observed common phosphoproteins in both of the considered treatment conditions relative to the corresponding expected random number as the distance metric. Smaller values of the inverse indicate larger overlaps, and thus, similar phosphoprotein sets. The different treatments segregated into two main classes, one containing treatments with the organic molecules mannitol and sucrose, the other containing the treatment with inorganic ions/salts NH^+^_4_, NO^−^_3_, and PO^3−^_4_ (Figure 1A).

**Table 2:** **Number of phosphoproteins found in common between two treatments**.

	**Mannitol**	**NH_4_**	**NO_3_**	**PO_4_**	**Sucrose**
Mannitol	157 (4.71)	3 (2.10)	5 (2.25)	13 (3.87)	13 (1.59)
NH4	3 (2.10)	70 (0.94)	19 (1.00)	13 (1.72)	5 (0.71)
NO3	5 (2.25)	19 (1.00)	75 (1.07)	22 (1.85)	8 (0.76)
PO4	13 (3.87)	13 (1.72)	22 (1.85)	129 (3.18)	9 (1.31)
sucrose	13 (1.59)	5 (0.71)	8 (0.76)	9 (1.31)	53 (0.54)

The number in parentheses is the expected common number of proteins in each pair of treatments based on the PhosPhAt database.

#### Dynamics of stimulus induced phosphorylation

Next, we included the dynamic information into the comparison of substance-induced phosphorylation events by focusing first on the qualitative change patterns across time. The *Change pattern* describes qualitatively how phosphorylation levels change from one time point to the next for a given phosphopeptide [increase, decrease, or unchanged relative to defined thresholds (see Materials and Methods), File S1]. The clustering of the different treatment conditions based on the change pattern statistic associated with all detected, nutrient-condition-specific phosphopeptides (Figure 1B) resembles the clustering obtained from the static presence/absence call (Figure 1A). Again, the inorganic nutrients (NO^−^_3_, NH^+^_4_, PO^3−^_4_) cluster together and are distinct from the organic solute treatments (mannitol and sucrose) with the latter exhibiting differing change patterns.

### Phosphorylation dynamics from a network perspective

So far, we have analyzed the available dynamic phosphorylation data in a statistically-descriptive and comparative fashion, in which the set of phosphoproteins were treated as independent entities. However, in the real biological system they are part of an interaction network and act in a whole-cell context. Thus, we investigated the set of phosphoprotein when embedded into the context of known protein–protein interactions and, secondly, we aimed to reconstruct the dynamic regulatory relationships between the identified phosphoproteins directly from the data by applying network inference methods.

#### Mapping phosphorylated proteins onto protein interaction networks

Since phosphorylation events are associated with protein–protein interactions via physical kinase-substrate encounters, we mapped the set of experimentally observed phosphoproteins onto the PIN of *Arabidopsis thaliana* (Dreze et al., 2011). This provided (a) context for those proteins that were observed in our dataset but without detecting their interaction partners, and (b) allowed us to investigate the specific role of phosphoproteins within the global network of physical interactions. We used AtPIN (Brandão et al., 2009) as a large-scale reference PIN, which, in addition to experimentally identified interactions, also includes many predicted interactions based on annotation transfer.

**Figure 2:**
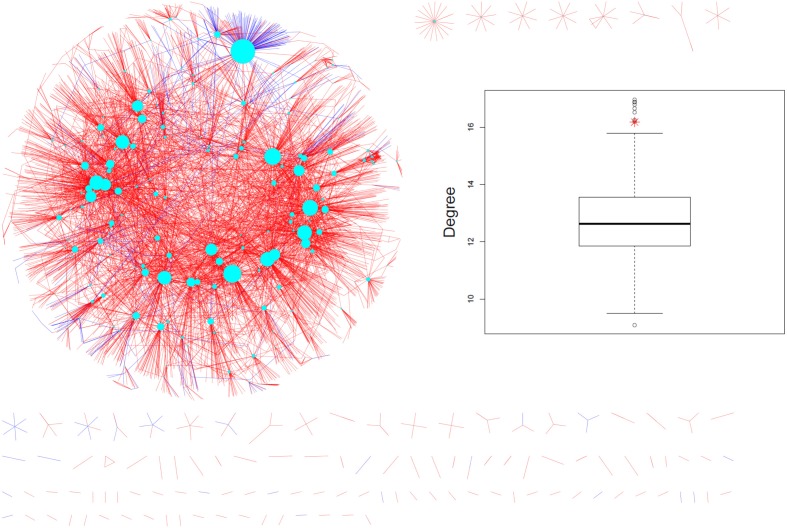
**Measured phosphoproteins mapped onto the AtPIN (june_2010 version).** The cyan nodes represent the phosphorylated proteins detected in the experiments and the size of the node is proportional to its degree. Coloring of the edges reflect the nature of the evidence such as experimental (blue color) or predicted (red color). The experimentally detected phosphoproteins and their direct interaction partners are shown only. The insert shows the real average degree of measured phosphoproteins shown in red star and average degrees of 1000 random protein sets from AtPIN with the same size to the measured phosphoproteins shown in black circle).

When mapped onto the AtPIN (Figure 2), the experimentally identified phosphoproteins were found to possess significantly higher interaction degrees [median (mean) of 6 (16.18)] compared to all other proteins [median (mean) of 4 (12.69), *p*-value = 1E-04 according to the non-parametric Mann-Whitney rank sum test (MWT)]. As our dataset represents only a small fraction of all experimentally identified phosphoproteins in Arabidopsis, we extended the PIN-degree comparison to all phosphoproteins contained in PhosPhAt (Durek et al., 2010) and compared them to all other proteins in the AtPIN. Again, phosphoproteins were found to engage in more physical interactions [median (mean) of 5 (15.28)) than non-phosphorylated proteins median (mean) of 3 (12.05), *p*-value_MWT_ = 0]. The average degree of phosphorylated proteins was found significantly higher also compared to a series of 1000 same-sized random protein sets drawn from the AtPIN (Insert in Figure 2).

We checked whether protein abundance may be a confounding factor in our analysis. While significant, no relevant positive correlation was observed between degree and reported protein abundance (Piques et al., 2009) with a Spearman rank correlation of *r* = 0.086 (*p* = 0.0001). Thus, protein abundance associated with an increased likelihood of experimental detection and possible increased degree cannot explain the observed degree differences. Thus, phosphoproteins appear to play a central role in the network of physical interactions between the proteins in Arabidopsis.

#### Network inference using a novel scoring scheme

When reconstructing networks from OMICS data, it is challenging to evaluate the accuracy of the employed reconstruction method without availability of suitable benchmark networks. Since the dataset studied here comprises only five time points, network inference is particularly challenging as the statistical power is low. We applied two scoring schemes (see Materials and Methods) that employ prior knowledge to help identify the most suitable inference method. The first method relied on subcellular localization information and scored predicted interactions by how likely they are based on the general observed frequency of interactions between proteins from the same or different compartments (SLPF-score, see Materials and Methods). The second scheme used the semantic similarity method (Wang et al., 2007) and judged predicted interactions based on the similarity of the respective Gene Ontology annotation terms as interacting proteins are likely involved in the same biological process and function.

A range of different reconstruction methods were tested and ranked based on the two scoring schemes. The applied inference methods included relevance based methods (Pearson correlation, first order partial correlation, full order partial correlation), mutual information based methods (MI), graphical model methods (Dynamic Bayesian network, DBN). Based on the two scoring schemes, Pearson correlation performed better than partial correlation (Table 3). The relevance based methods (correlation-based methods) also performed better than model- and mutual information-based methods, probably explained by the limitations of the dataset (short length and noise). It should be noted that the GO annotation used in the semantic similarity score was possibly based in part on protein interaction information as available in AtPIN as well so that the two applied scorings schemes are not entirely independent.

**Table 3:**
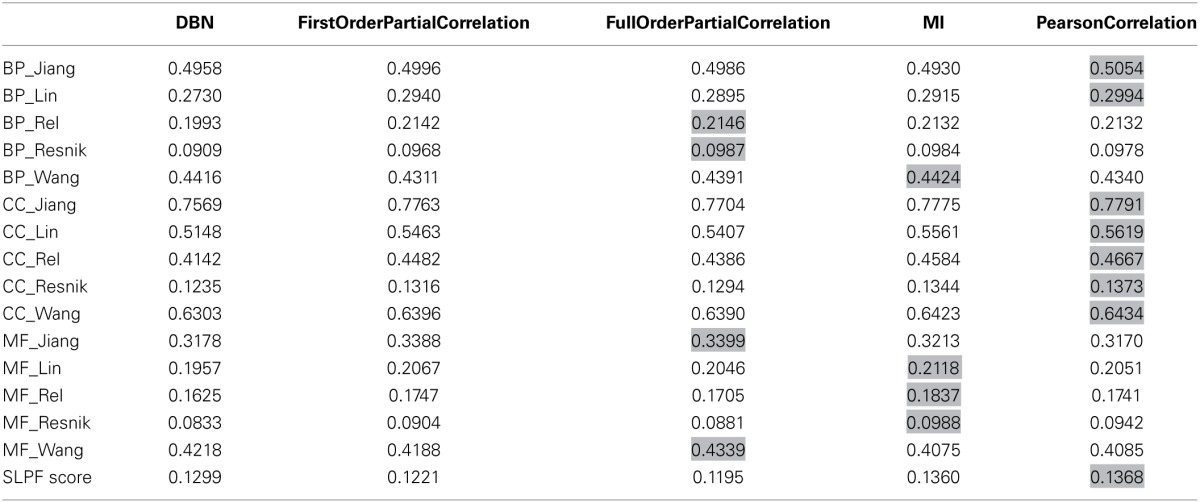
**Accuracy scores of the reconstructed networks with same size generated by different methods using the subcellular location pair frequency (SLPF) metric and semantic similarity of gene ontology terms (BP, CC, and MF)**.

“^*^_Jiang”, “^*^_Lin”, “^*^_Rel”, “^*^_Resnik” and “^*^_Wang” represent different semantic similarity metric as introduced in Wang et al. (2007).

Larger score values correspond to networks with higher confidence. The gray shaded values identify the highest score within each scoring metric.

DBN, dynamic Bayesian network; FirstOrderPartialCorrelation, first order partial correlation; FullOrderPartialCorrelation, full order partial correlation; MI, mutual information; PearsonCorrelation, Pearson correlation. The SLPF scoring scheme was based on AtPIN.

Based on the overall-performance of the various inference methods as judged by the two scoring schemes (Table 3), we decided to use the Pearson correlation-based method to produce the final network of phosphoproteins derived from the available data. As a disadvantage compared to GGMs or other graphical model methods, Pearson correlation-based inference methods result in undirected networks. In order to infer cause-effect relationships (i.e., directed networks), we assigned directionality to every edge as introduced in (Schäfer and Strimmer, 2005; Opgen-Rhein and Strimmer, 2007). Here, directionality assignments are based on the log ratio of partial variance between the two nodes connected by an edge (see Materials and Methods).

**Figure 3:**
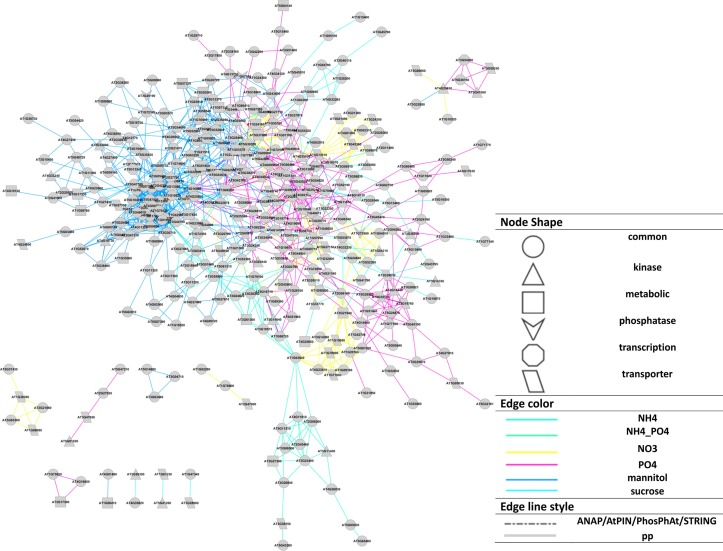
**Cytoscape representation (Smoot et al., 2011) of the reconstructed network based on Pearson correlation (*p* ≤ 0.02).** Node shapes represent protein functions, edge colors represent the treatments source for each interaction, edge line style represents if an interaction exists in available database (“pp” means the corresponding interaction does not exist in available PIN databases). A force-directed layout was used.

The different nutrient/osmotic treatments resulted in separate subnetworks connected, and thus merged, via central proteins involved in compound uptake and signaling (Figure 3). For example, plasma membrane ATPases (AT2G18960, AT4G30190), and water channel PIP2E (AT2G39010) are regulated by phosphorylation and are involved in all different treatments. Among the signaling proteins, calmodulin binding protein (AT1G74690) was detected involved in the organic molecule responses (mannitol and sucrose), but not with the inorganic salts. Three different nutrient responses involved the MAP kinase MKK2 (AT4G29810). In all treatments, phosphorylation changes were observed for the metabolic enzyme glutamate synthase (AT1G66200), which was reported to integrate responses for changes in carbon, nitrogen, and phosphate status through alterations in phosphorylation status (Oliveira and Coruzzi, 1999; Lima et al., 2006).

The resulting final network consisting of the superset of all interactions inferred for all individual nutrient conditions separately and merged via the bridging (jointly occurring) phosphoproteins (Table S1, File S2) was used in all subsequent analyses.

#### Topology characteristics of the reconstructed integrated network

**Figure 4:**
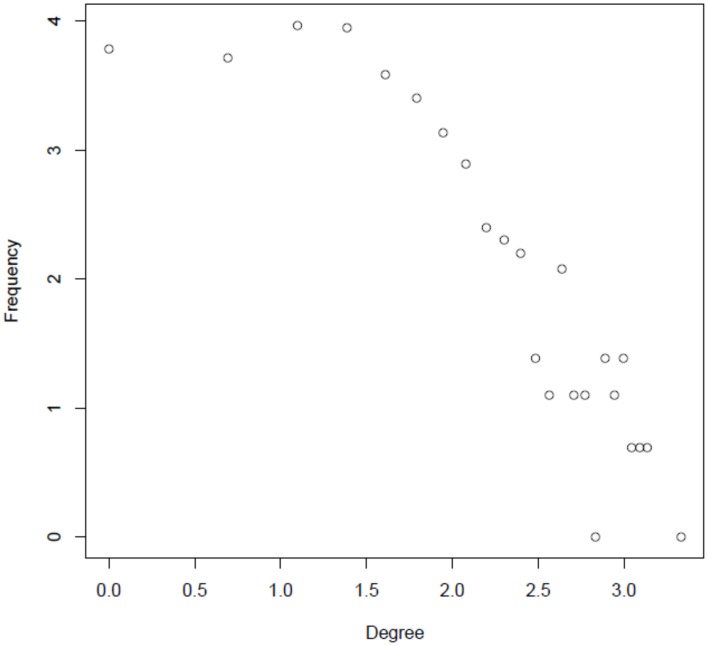
**Degree distribution of the reconstructed integrated network in a log–log plot (natural logarithm)**.

A primary goal of this study was to characterize the regulatory interactions between phosphoproteins that are dynamically activated or inactivated in response to changes of nutrient supply. We addressed this question by using various parameters that are classically used to describe complex interaction networks (Klemm and Bornholdt, 2005). Node degree frequencies in the reconstructed network appear to be relatively constant up to degree values of 4–5 (log_*e*_4 = 1.38). Above this value, the degree distribution follows a power law distribution (Figure 4), a commonly found characteristic of molecular interaction networks (Yook et al., 2004; Albert, 2005). Nodes with greater degree values are becoming increasingly unlikely, but the few nodes with high degree levels may serve as central processing hubs (Table S2). In particular, nucleic acid binding proteins, such as transcription factors were found among the proteins with high degree. This class of proteins has previously been found to contain high numbers of clustered phosphorylation sites in so-called phosphorylation hotspots (Riaño-Pachón et al., 2010; Christian et al., 2012).

**Figure 5:**
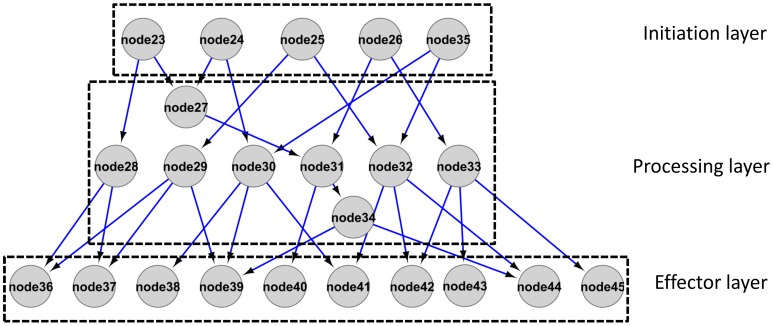
**Schematic representation of a network architecture with broadening scope (“information dissemination topology”) as presented from the current dataset in File S3 and S4**.

For the reconstructed integrated network, directionality was determined based on the log ratio of partial variance as introduced in Schäfer and Strimmer (2005) and Opgen-Rhein and Strimmer (2007). Given the applied threshold (see Materials and Methods), directionality was assigned to 343 out of 1067 total interactions. In the following, we further inspected the directed interactions as candidate cause-effect relationships only. We defined proteins with no in-degree but out-degrees of one or greater as the “initiation” layer of the network as the signaling cascade is initiated at this level given the network. Proteins with one or greater in- and outgoing edges form the “processing” layer. Proteins with only incoming but no outgoing edges (out degree zero) constitute the “effector” layer as the signal processing ends at those nodes (Figure 5). In the reconstructed network, the numbers of nodes in the initiation/processing/effector layers were identified as 75/98/104, respectively. Thus, the network “fans out” with higher numbers of processing proteins than initiators and still more effectors than processing proteins (Cytoscape file as File S3, list in File S4). Accordingly, the in-degree [median (mean) of 1(1.7)] is significantly lower than the out-degree [median (mean) of 2(2.1)] for processing layer nodes (Mann-Whitney test, *p*-value: 0.02). Taken together, the reconstructed network conforms to a topology in which the information appears disseminated to an increasingly broadened set of phosphoproteins.

In the “initiation” layer, we observed an overrepresentation of plasma membrane proteins (*p* = 0.011, Fisher Exact test), compared to processing and effector layers. By contrast, the “effector layer” is particularly enriched for proteins with cytosolic location (*p* = 0.0012, Fisher Exact Test) and proteins with nuclear location (*p* = 0.0048, Fisher Exact Test). Thus, external stimulation induces signaling processes at the plasma membrane that are relayed to cytosolic and nuclear effectors. The enrichment for transcription-related proteins was highest among the effector proteins (*p* = 0.059, Fisher Exact Test). Interestingly, also among the effectors, a large number of kinase proteins (*p* = 0.0014, Fisher Exact Test) was found, indicating that some of the observed effectors may still be involved in further “processing”, but their targets were not captured within the time window analyzed in this study (30 min).

In general, 45% of the proteins of the initiation layer displayed a maximum change point of phosphorylation at 3 min. In contrast, the majority of proteins in the processing and effector layer showed maximum change points in phosphorylation at later time points, such as 10 min (41% for processing, 44% for effectors). Thus, the initiation layer in all treatments largely does refer to the “early” responses particularly involving plasma membrane proteins, while processing and effector layer are enriched in proteins with “late” responses particularly located in the cytosol or nuclear compartments as has already been suggested for nitrogen treatments (Engelsberger and Schulze, 2012).

#### Phosphorylation motifs in the layered network

**Figure 6:**
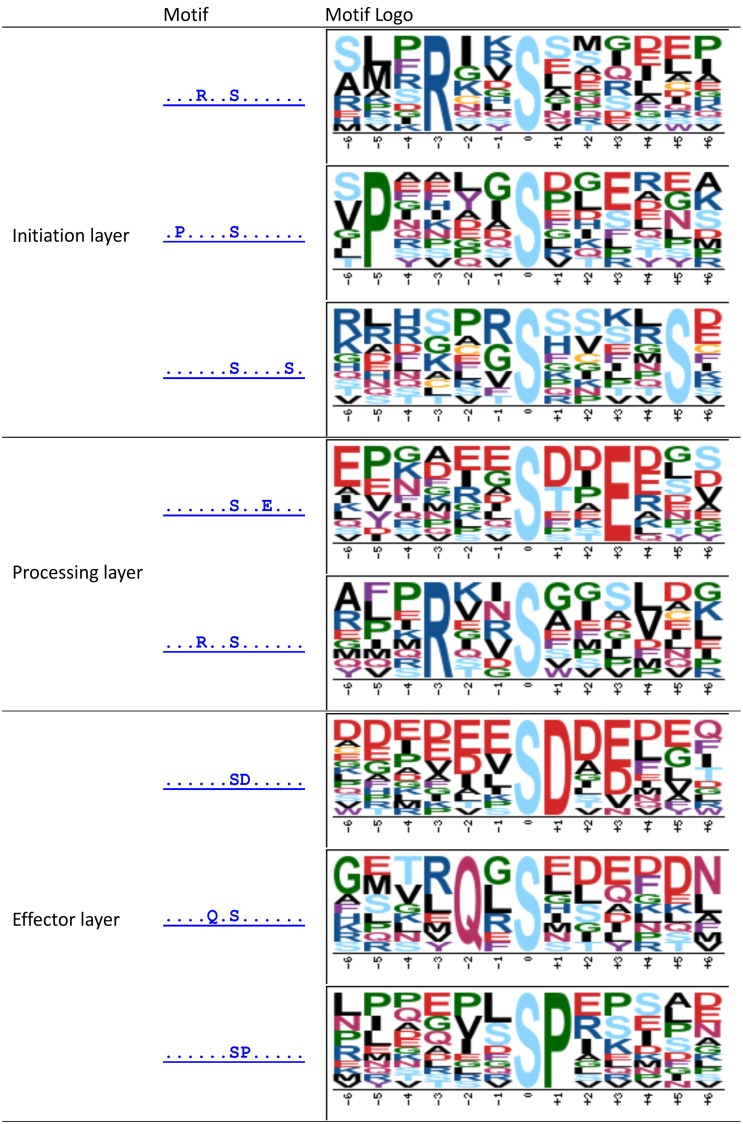
**Enriched motifs of phosphopeptides in different layer of the reconstructed network.** Motif-x was used with *p*-value threshold 0.01, the occurrence threshold was set to 10.

Based on the layered functional topology of the reconstructed network, we checked for the presence of layer-specific phosphorylation motifs (Figure 6). The initiation layer was found to contain motifs with basic amino acid residues RxxS, which are likely targets of calcium dependent protein kinases. The double-S motif is common among membrane proteins, such as aquaporins and was identified as a common motif of a plant receptor kinase (Wu et al., 2013). The PxxxxS motif is yet uncharacterized. The processing layer was also enriched in the basic RxxS motif again possibly involving members of the CDPK/SnRK family. Interestingly, in the effector layer, we found a significant (*p*-value = 2.32E–05, Fisher's exact test) over-representation of SP motifs, which were shown to be targets for MAP-Kinases. Thus, the identified phosphorylation motifs also reflect the functional layout of the reconstructed network from targets of membrane receptor kinases and CPDKs or PKA in the initiation layer to MAP-Kinase targets in the effector layer. The kinase specificity motifs with acidic amino acids, such as SxxE, QxS and acidic motifs SxxE and SE are likely targets of casein kinase II. These acidic motifs are particularly represented in the processing and effector network layer. These acidic casein kinase II motifs were also particularly present for the protein kinases AT4G08170, AT4G10730, and AT4G38470 (Figure S1), which were the kinases with highest degree in our reconstructed network.

## Discussion

In this study, the dynamics of nutrient-induced protein phosphorylation was investigated. The tested inorganic nutrients (NH^+^_4_, NO^−^_3_, and PO^3−^_4_) provoked distinctive responses compared to the organic solutes (sucrose, mannitol) with regard to the participating phosphoproteins/peptides and the dynamic change patterns. Based on the time series of peptide phosphorylation levels, an integrated phosphorylation signaling network was reconstructed using a newly designed scoring metric for the selection of the most appropriate network inference method. In general, the topology of the inferred networks corresponds to an information dissemination architecture in which the phosphorylation signal is passed on to an increasing number of processing proteins stratified into an initiation, processing and effector level layer. Specific phosphorylation peptide motifs associated with the three distinct layers were identified indicating the action of layer-specific kinases.

Due to limitations on experimental capacity as well as mass spectrometric instrument time, the time series data used in this study was rather short, but still constituted one of the largest phosphoproteomic time series studied in plants so far. Nonetheless, with only five time points available, reliable network inference is challenging as the statistical power needed to infer interactions between proteins is low. As protein phosphorylation levels were found to change considerably, we conclude that the probed time interval (30 min) was chosen adequately to capture the initial signaling events even though the presence of protein kinases in the effector layer suggests that the dynamics is still ongoing at the end of the measuring interval. Apart from the limitations with regard to temporal resolution and time interval, the available phosphoprotein set can be considered limited also with regard to phosphoprotein coverage. (Phospho)proteomic data sets collected still suffer from being incomplete due to slow instrument acquisition cycles resulting in lower proteome coverage than is commonly achieved in gene expression profiling using whole-genome microarrays. Particularly, the requirement for quantitative values for all five time points reduced the current data set from about 1000 identified phosphopeptides to the core dataset of approximately 550 peptides with intensity values from label-free quantitation. Out of these, only for 24 peptides full time course data were available from different biological replicates. Nonetheless, we probed whether the underlying data are sufficiently robust by comparing the time profiles of the available repeat measurements for the same peptide, which we expect to be similar, to profile comparisons across different peptides. Indeed, the same-peptide-repeat profiles are much more similar to one another (average Pearson correlation coefficient, *r*_avg_ = 0.32) than peptide profiles associated with different peptides (*r*_avg_ = 0.02, *p*_Mann−Whitney_ = 2.2*E* − 05). Given the data limitations and technical challenges, the success of network inference attempts critically depends on the selected computational inference method. We performed additional analyses to test the validity of the detected relationships between phosphoproteins.

### Evaluation of network inference methods

We systematically benchmarked a range of different methods by judging their ability to reproduce networks that are consistent with prior biological knowledge. In particular, we used subcellular localization information and consistency of gene ontology annotations to assess the merit of the networks obtained by the various tested methods. We found that relevance-based networks such as linear Pearson correlation and graphical Gaussian method based on regularized partial correlation calculation, and designed initially for large scale gene regulatory network reconstruction, worked best. In a similar study also involving a five-timepoint phosphoproteomic study in growth-factor stimulated HeLa cells, Pearson correlation was also used successfully in the network reconstruction of SILAC-based quantitative data (Imamura et al., 2010). In general, given the data set limitations, simpler methods; i.e., methods with fewer parameters, may prove superior compared to methods needing large datasets for model and parameter fitting.

In principle, time series data allow detecting cause-effect relationships via time-delay based correlations or using more advanced concepts such as Granger causality (Walther et al., 2010). However, with only five time points available here, such approaches were not applicable. Nonetheless, as those methods bear great potential, efforts should be undertaken to sample as many time points as possible.

We used protein subcellular localization and gene ontology in the network reconstruction method performance assessment. Intuitively, this information could also be used directly during the network inference process. In fact, considering prior information such as known interaction from online databases has been used as additional weighting information in gene regulatory network or signaling network reconstruction efforts integrating prior interaction information in the framework of Bayesian networks (Lo et al., 2012; Pei and Shin, 2012). The assumption that interacting proteins are more prone to interact in the same subcellular compartment (similar functions or related biological pathway) has been used in protein function annotation studies (Van Noort et al., 2003).

Utilizing target-specific motif information as a type of prior information also bears potential for use in phospho-signaling network reconstruction. Based on the assumption that targets of a particular kinase have conserved motifs, kinase-target relationships may be predicted from sequence alone (Yaffe et al., 2001). However, due to the still incomplete understanding of known kinase-substrate motif information for many plant specific kinases, we did not use this information in our approach. Efforts to infer kinase-substrate interaction networks using existing information from large-scale protein–protein interaction studies as prior information may contribute toward expanding the knowledge of kinase-target relationships in the future (Linding et al., 2007; Xue et al., 2008; Song et al., 2012; Newman et al., 2013).

### Support for the validity of the reconstructed network

The reconstructed network was compared to available protein–protein interaction databases (Table 4). Eighteen of the predicted edges are also reported in the various PIN-resources as direct physical interactions compared to only seven agreements on average for randomized networks of the same size (*p* < 0.001, same number of edges, 1000 randomization runs). In addition, within the experimental phosphopeptide data set, we found higher frequencies of positive correlations between phosphopeptides compared to randomized data sets (Figure S2) suggesting that a systematic signals are contained in the data. While this signal may also originate from overall technical biases, the former result of increased numbers of confirmed correlations via protein–protein interactions supports the view that the detected signal stemmed primarily from the underlying biological processes. The edges identified in our regulatory network as well as in protein–protein interaction networks include interaction in metabolic pathways (PGM—F2KP; 14-3-3—CINV1), ribosomal proteins (RPP1A—RPP2B, RPP2A—RPP3A) as well as the plasma membrane ATPases (AHA1—AHA2). Although these edges are not kinase-substrate pairs, they are likely to be regulated by consecutive events in common pathways. Particularly for ribosomal proteins and the plasma membrane ATPase, regulation by phosphorylation has been described (Fuglsang et al., 2003; Hummel et al., 2012).

**Table 4:** **Common interactions between the reconstructed network and online PIN databases**.

**Partner A**	**Partner B**	**Source**
AT1G23190	AT5G51820	AtPIN/STRING
AT1G23190	AT1G07110	STRING
AT5G38480	AT1G35580	ANAP/STRING
AT2G27720	AT4G25890	ANAP/STRING
AT5G61780	AT5G07350	STRING
AT1G08420	AT4G03080	AtPIN
AT2G18960	AT4G30190	AtPIN/STRING
AT3G48740	AT2G18960	STRING
AT2G18960	AT1G57990	STRING
AT1G01100	AT2G27710	AtPIN/STRING
AT1G15690	AT1G78900	STRING
AT4G35100	AT2G39010	AtPIN/ANAP/STRING
AT3G09200	AT1G31340	ANAP
AT3G52400	AT1G59870	ANAP/STRING
AT5G45510	AT2G27710	STRING
AT5G37600	AT1G66200	AtPIN
AT3G14350	AT2G39010	STRING
AT1G22280	AT2G40770	STRING

PIN-databases: AtPIN (Brandão et al., 2009), ANAP (Wang et al., 2012a), and STRING (Franceschini et al., 2013).

Nonetheless, most protein pairs predicted in this study are not reported as direct interactions in the various PIN databases. Aside from acknowledging the obvious possibility that those predicted interactions may be false-positives, we investigated whether the novel, but uncorroborated node pairs may constitute plausible interactions in the sense that they are predicted to occur in a local PIN-environment. We mapped the predicted node pairs onto the integrated PIN (combination of AtPIN, ANAP, and STRING) and computed the shortest path lengths between them. Compared to node pairs drawn at random from the available phosphoprotein set, we found that connected pairs, though not interacting directly, frequently correspond to local interactions as their average shortest path length is significantly smaller (mean = 4.56) than for random pairs (1000 repeats, mean = 4.95, stdev = 0.12, *p* < 0.001). Thus, in many cases, the interactions detected by our time-resolved phosphorylation network inference approach appear to be indirect regulatory interactions for which immediate protein partners are not present in the data set used here. Alternatively, they may correspond to altogether novel interactions as the predicted interactions may be specific for the particular conditions investigated here, which may not have been captured by other protein–protein interaction studies. For example, several regulatory interactions between protein kinases (e.g., MKK2) and DNA-binding proteins (e.g., RGD3) or transcription factors were identified in the reconstructed network, which were not present in the protein–protein interactions data set. Moreover, the regulatory interactions shown here seem to “shortcut” direct interaction paths.

While we intentionally chose a reconstruction method that best reproduced prior biological information, the actual information itself was not used during the reconstruction process. Furthermore, the protein–protein interactions did not enter directly into the performance assessment metric, but rather the probability of protein–protein encounter based on subcellular localization. Thus, the reported results in support of the validity of the predicted interactions go beyond a circular argument.

### Nutrient-induced phospho-signaling in arabidopsis

Even though the detected phosphoprotein sets in the different nutrient treatments overlapped more than expected by chance (Table 2), the generated networks for the individual treatments did not result in common directed interactions between them and could only be merged based on jointly occurring phosphoproteins. Thus, with regard to interactions, the treatments resulted in disjoint sets. Of course, the above mentioned data set limitations may explain this observation. In addition, because the number of possible directed interactions scales with the square of the number of phosphoproteins and, furthermore, the resulting networks are sparse, overlaps between them are less likely than between the proteins sets themselves. If, on the other hand, the result of disjoint interaction sets is taken as the truth, it can be concluded that the different nutrient or osmotic stimuli trigger different signaling cascades. Particularly, it is known that the mere osmotic component (i.e., in mannitol treatment or also sucrose) is primarily perceived through a histidine-kinase sensory system (Urao et al., 1999) which inherently differs from transporter-based sensing systems in nitrogen acquisition (Ho and Tsay, 2010). It is interesting to note that the phosphoproteins commonly found in several different treatment conditions (Table 2) preferentially fall into the processing level layer [55% on average, significantly more than expected by chance (*p* = 1.2E–4, binomial distribution processing layer vs. others accounting for layer set sizes as well)]. This supports the network topology with a specialized smaller number of initiators that induce the specific response but feed into common proteins with information processing role. On level of the effectors, again larger specificity could be observed regarding the different treatments.

In biological signaling cascades, an increase of interaction space for downstream kinases has been observed before. An example is the MAP-Kinase cascade, in which the final effector kinase, the MAP-Kinase (MPK) is activated by a MAP-Kinase-Kinase (MKK), which in turn is activated by a MAP-Kinase-Kinase Kinase (MAP3K). In protein array experiments identifying substrates to each of the different levels in the MAP-Kinase cascade, largest numbers of interactions (Popescu et al., 2009) were found with the MPKs (570 substrate candidates) while “incoming” interactions of MPKs with the activating kinases MKK resulted in far less confirmed interactions (9 MKKs). The MPK signaling pathway is particularly involved in responses to abiotic stress such as salt stress. Thus, the observed links to the MPK cascade could particularly be an osmotic response and was especially observed with the mannitol treatment.

A great diversity was also observed in the plant receptor kinases, many of which feed into soluble kinase signaling pathways (Osakabe et al., 2013). This receptor diversity particularly reflects the plant's potential to respond to different stimuli with only a small number of receptor/co-receptor pairs being activated by the particular signal and serving as initiators for downstream cascades. Therefore, the topology observed in our reconstructed network is indeed reflected in biological signaling networks with also receptor kinase as members of the initiation layer.

In our analysis, only proteins with phosphoproteomic profiles consisting of five time points were included for network reconstruction. This greatly reduced the data set from about 1000 of identified phosphopeptides in the original studies to only approximately 500 peptides studied here. By imposing directionality, the layered network only included 277 phosphoproteins. Important players in nutrient signaling include the Snf1-related kinase family (SnRK) (Shin et al., 2007) and a central growth regulator TOR (Caldana et al., 2013), which was also shown to interact with nutrient responses. Although members of these kinase families were found to be phosphorylated in response to nitrogen changes (Engelsberger and Schulze, 2012), the data density was not high enough to record full time profiles for these proteins and thus they could not be included in this work. This indicates that key responses do occur at individual time points only and these cases cannot always be clearly distinguished from missing data points due to data-dependent acquisition by mass spectrometry (Schulze and Usadel, 2010). Future challenges lie in efficiently including and biologically weighting single occurrences within the signaling network context.

## Conclusion

A primary goal of this study was to characterize the regulatory interactions between phosphoproteins that are dynamically activated or inactivated in response to changes of the nutrient and organic-solute challenges supply. To address this question, we used methods of classic statistical interaction network description and developed a novel scoring metric for inferred networks. Our main findings suggest a hierarchical architecture of regulatory phosphorylation networks with proteins involved in signal initiation, signal processing, and an effector layer. An increasing number of proteins in downstream hierarchical layers of the network were found which is consistent with information processing features typically observed in phosphorylation cascades. Our work demonstrates that even with short time course data sets, network inference methods are applicable for the reconstruction of information processing networks.

## Materials and methods

### Experimental data

Time series phosphoproteomic data were obtained from two published data sets involving sucrose and mannitol treatment (Niittylä et al., 2007), as well as nitrate and ammonium treatment (Engelsberger and Schulze, 2012), or phosphate treatment. The dataset is publicly available in the PhosPhAt database (Durek et al., 2010). Briefly, in all treatments, the experimental design involved starvation of 14-day old Arabidopsis seedling for a particular nutrient and resupply of the respective nutrients for various time periods (3, 5, 10, 30 min) after which samples were taken. The starved seedlings served as controls for that particular nutrient (0 min). Protein extracts were fractionated into plasma membrane and soluble proteins before tryptic digestion and enrichment of phosphopeptides over TiO2 or IMAC. Phosphopeptides were subsequently analyzed by LC-MS/MS on an Orbitrap mass spectrometer. Label-free quantitation for all experiments was carried out based on ion intensities of phosphopeptides and involved protein correlation profiling to obtain quantitative values for precursor ions without fragmentation spectra.

### Expected number of phospho-proteins common to pairs of treatments

For a given pair of treatments *T*_1_ and *T*_2_, the expected random number of phosphopeptides observed in both treatments was calculated as *N*_*T*_1__ · *N*_*T*_2__/*N* assuming independence of the individual observations with *N* referring to the total number of Arabidopsis phosphoproteins (5237, downloaded on 10.20.2011) contained in the PhosPhAt database (Durek et al., 2010), and *N*_*T*_1/2__ referring to the number of phosphoproteins observed in the respective treatment conditions.

### Hierarchical clustering of phosphoprotein sets

Using a dissimilarity metric (the inverse of the ratio between the actual number and the randomly expected number of phosphoproteins observed jointly), the nutrient treatments were clustered using hierarchical clustering (complete linkage).

### Data set normalization

For each treatment, the original intensity values were first log-transformed (natural logarithm). Afterwards, the median value of each time point across all peptides was subtracted from every individual value for a particular peptide and time point.

### Detection of significantly changed peptide phosphorylation levels between consecutive time points

After normalization, phosphorylation of a particular phosphopeptide was considered changed, if the difference *d* with *d* = *a*[*t*] − *a*[*t* − 1], *t* ≥ 2, where *a*[*t*] represents the phosphorylation level at time point *t*, was outside of a specified threshold range set to [−1.71, 1.64] for down- and upregulation, respectively. The threshold criteria selection was based on the observed mean and standard deviation of *d* across all intervals and across all peptides for all treatments with the interval of no-change determined as [mean(*d*) − std(*d*), mean(*d*) + std(*d*)].The dynamics of phosphorylation level of a particular P-site was described qualitatively as a succession of “increase,” “decrease,” and “no change” (relative to thresholds defined above) events and referred to as the *change pattern*.

Note that despite phosphorylation being a binary on/off event for any given phosphorylation site, phosphorylation levels were considered as continuous variables as the measured experimental phosphorylation levels resulted from an average across many protein copies in the sample with most likely not fully coordinated on/off phosphorylation events.

### Hierarchical clustering of change patterns

Each peptide time series was translated into a succession of four qualitative changes of “increase,” “decrease,” or “no change” based on the thresholds explained above. For each treatment, a frequency vector with length 81 was calculated corresponding to the maximal number of theoretically possible change pattern series (3^4^ = 81). Each element in the vector represents the proportion of peptides following the corresponding change pattern in a particular treatment. Using the Euclidean distance of the frequency vectors for each pair of treatments, the nutrient treatments were clustered using hierarchical clustering (complete linkage).

### Protein interaction networks

PIN data was obtained from the public resources AtPIN (Brandão et al., 2009), as of October 2011, comprising 96,821 interactions between 15,163 *Arabidopsis* proteins including both experimentally verified and predicted interactions. In the assessment of reconstructed interactions, several other online PIN databases [PhosPhAt (Zulawski et al., 2013), ANAP (Wang et al., 2012a), and STRING (Franceschini et al., 2013)] were also used. These databases also collect existing evidence for the potential protein interaction in Arabidopsis.

### Network inference methods

Various methods of network inference methods were applied including correlation based methods (Peng et al., 2009) (Pearson correlation [PearsonCorrelation], partial correlation[FirstOrderPartialCorrelation], mutual information based method [MI] (Sales and Romualdi, 2011), graphical model methods (Schäfer and Strimmer, 2005; Lèbre, 2009) (Dynamic Bayesian network [DBN], graphical Gaussian model [FullOrderPartialCorrelation]). The abbreviation in square brackets are also used in Table 3. The R package *parmigene* was used for the mutual information based method. The R packages *space* was used for partial correlation based methods. The R package *G1DBN* was applied for dynamic Bayesian network inference. The R package *GeneNet* was used for the graphical Gaussian model method.

Several of the employed methods require setting threshold parameters, e.g., a minimum correlation coefficient and/or associated *p*-value of correlation. Evidently, less strict thresholds result in larger networks. In order to compare methods between each other based on obtained networks of the same size, networks were pruned to a common size of 3^*^N (*N* is the number of measured phosphopeptides) by eliminating edges and associated nodes in decreasing score order; i.e., high-confidence edges were retained. The size 3^*^N was chosen as molecular interaction networks often exhibit an average degree of 2–4 (Werhli, 2012). Networks were first reconstructed for each nutrient treatment condition separately and then merged into a single, final network as the superset of all individual predicted interactions.

### Network inference method performance assessment metric

In order to evaluate the accuracy of the reconstructed networks, we devised a novel scoring metric that is based on the subcellular location of proteins. Predicted interactions between proteins were scored by how likely they are based on prior knowledge of physical interactions between proteins from different or the same cellular compartments. For all pairwise physical interactions contained in AtPIN, the subcellular localization of the two interacting proteins as reported in SUBA (Heazlewood et al., 2007) was determined resulting in a comprehensive frequency table of physical interactions between proteins from all compartments termed the subcellular location pair frequency (SLPF, File S5). The corresponding subcellular location pair frequency network is shown in Figure S3, the width of the edge is proportional to its frequency. For example, it is very likely that two interacting proteins are both from the nucleus as the corresponding edge (arc to itself) has a large width. By contrast, direct physical interactions between proteins located in the nucleus and in the endosome are less likely. Hence, network inference methods can be compared to each other by summing up the SLPF-score computed for all predicted set of interactions dividing subsequently by the number of predicted interactions to account for different network sizes. Better methods will be associated with higher SLPF scores.

In addition to the SLPF-score, we also used the Gene Ontology (GO) semantic similarity method (Wang et al., 2007), which includes not only compartment component, but also molecular functions and biological pathway, as a metric to assess the validity of the reconstructed networks. Here, interactions are scored by how similar the GO annotations of proteins predicted to interact are. Again, scores are summed up for all predicted interactions and divided by their number.

### Directionality of correlation-based interactions

After reconstructing the network based on Pearson correlation, we used the method introduced in (Schäfer and Strimmer, 2005; Opgen-Rhein and Strimmer, 2007) to calculate the directionality of each interaction. First, the shrinkage estimate of correlation was computed by function “cor.shrinkage” in R package “corpcor.” Secondly, concentration matrix was calculated as the inverse of the correlation matrix by function “solve” from R package “base.” Finally, the partial variance was obtained as the reciprocal of diagonal element of the concentration matrix, and then the ratio of partial variance was calculated correspondingly. The direction of the arrows points from the node with the larger standardized partial variance (the more “exogenous” variable) to the node with the smaller standardized partial variance (the more “endogenous” variable). The directionality threshold value was set to a log-ratio of partial variances of ±0.002. The threshold was obtained as the average top and bottom 25 percentile of log-ratios across all five treatment conditions.

As networks were reconstructed separately for every treatment, conflicts of directionality assignments are, in principle, possible. However, no directed edge between any two proteins was found common to any pair of treatments.

### Network properties

In undirected networks, the degree of a node *n* is the number of edges linked to *n* (Assenov et al., 2008). The node degree distribution gives the number of nodes with degree *k* for *k* = 0, 1, …. In directed networks, the in-degree of a node *n* is the number of incoming edges and the out-degree is the number of outgoing edges.

### Functional term enrichment analysis

Fisher's exact test was used in the functional and localization enrichment analysis based on the available Gene Ontology annotation information from TAIR (version 10) (Lamesch et al., 2012), MAPMAN annotation (Thimm et al., 2004) and SUBA subcellular localization information. Specific GO-function terms associated with phosphorylation processes were selected including “common” (=all other categories), kinase, metabolic, transporter, and phosphatase. Selected GO-localization terms were tested to detect preferences with regard to subcellular localization including the terms nucleus, Golgi, mitochondrion, endoplasmic reticulum, cytosol, extracellular, vacuole, plasma membrane, plastid, peroxisome, cytoskeleton, cell plate, endosome.

### Semantic similarity

The semantic similarity of the gene ontology (GO) terms associated with two genes was calculated based on the “Wang“ measure (Wang et al., 2007), which aggregates the semantic contributions of their ancestor terms (including this specific term) in the GO graph. The R package “GOSemSim” was used in the semantic similarity calculations (Yu et al., 2010).

### Subcellular localization information

Subcellular localization information of Arabidopsis proteins was obtained from the SUBA database (Heazlewood et al., 2007; Tanz et al., 2013).

### Phosphorylation-site motif detection

Motif-x was used in the phosphopeptide motif analysis, which is an iterative strategy to build successive motifs through comparison to a dynamic statistical background (Schwartz and Gygi, 2005). The phosphopeptide sequence was extracted with length 13 (the phosphorylation site was in the middle position). The *p*-value threshold was set to 0.01 and the occurrence threshold was set to 10 as the motif-x web service setting-up. The whole Arabidopsis proteome was selected as the background reference.

## Conflict of interest statement

The authors declare that the research was conducted in the absence of any commercial or financial relationships that could be construed as a potential conflict of interest.

## Supplementary material

Figure S1 **Phosphopeptide motifs of extracted from peptides associated with proteins which have an absolute correlation coefficient greater than or equal to 0.8 with selected kinases based on the respective phosphorylation level profiles.** The analysis was done for those kinases with at least 10 correlated proteins. Those proteins and their associated peptides were then interpreted as the kinase targets. motif-x (Schwartz and Gygi, 2005) was used with *p*-value threshold 0.01, the occurrence threshold was set to 10.

Figure S2 **Distribution of Pearson correlation coefficients associated with the actual experimental data (black) and randomized data (red lines, 1000 randomizations)**.

Figure S3 **Subcellular location pair frequency network derived from AtPIN and SUBA.** The width of each edge is proportional to its frequency. Cytoscape was used to visualize the network. Predicted interactions can be scored by how likely they are given the expected interaction frequencies displayed here.

File S1 **List of phosphopeptides including their dynamic change patterns.** In the “change pattern” column, “*N*” represents no change at corresponding time point compared to the previous one, “U” and “D” represents up-regulated and down-regulated, respectively.

File S2 **Cytoscape file of the joint reconstructed Pearson correlation network based on the different nutrient treatments**.

File S3 **Cytoscape file of directional network in layered hierarchical layout.** The log ratio of partial variance was set to 0.002 to obtain the directional network for interactions in the network of File S2 or Figure S3.

File S4 **List of phosphoproteins and their properties in the layered network.** “protein based layer” column shows the layer position for each protein in this directional network.

File S5 **Interaction frequency for each pair of subcellular localizations.** The subcellular localizations include nucleus, Golgi, mitochondria, endoplasmic reticulum, cytosol, extracellular, vacuole, plasma membrane, plastid, peroxisome, cytoskeleton, cell plate, endosome, and unclear.

File S6 **Commonly detected proteins among different pairs of treatments**.

Table S1 **Reconstructed network summary (number of nodes, number of interactions, and average degree) for each treatment, which was then combined into an integrated “super-“network**.

Table S2 **List of top 20 nodes (phosphoproteins) with highest degree in the reconstructed network**.
