# Supplementary material for: Reconstruction and analysis of nutrient-induced phosphorylation networks in Arabidopsis thaliana
Source: Front Plant Sci. 2013 Dec 24;4:540. doi: 10.3389/fpls.2013.00540 (PMC3872036; doi:10.3389/fpls.2013.00540)
Supplement: Figure S1 — Phosphopeptide motifs of extracted from peptides associated with proteins which have an absolute correlation coefficient greater than or equal to 0.8 with selected kinases based on the respective phosphorylation level profiles. The analysis was done for those kinases with at least 10 correlated proteins. Those proteins and their associated peptides were then interpreted as the kinase targets. motif-x (Schwartz and Gygi, 2005) was used with p-value threshold 0.01, the occurrence threshold was set to 10. [file DataSheet1.ZIP › supplementary table1.docx]

**Supplementary Table 1**. Reconstructed network summary (number of nodes, number of interactions and average degree) for each treatment, which was then combined to a super-network.

| Treatment | Number of Nodes | Number of interactions | Average degree |
| --- | --- | --- | --- |
| mannitol | 149 | 530 | 3.56 |
| NH4 | 54 | 100 | 1.85 |
| NO3 | 66 | 138 | 2.09 |
| PO4 | 125 | 260 | 2.08 |
| sucrose | 36 | 40 | 1.11 |
