# Supplementary material for: Reconstruction and analysis of nutrient-induced phosphorylation networks in Arabidopsis thaliana
Source: Front Plant Sci. 2013 Dec 24;4:540. doi: 10.3389/fpls.2013.00540 (PMC3872036; doi:10.3389/fpls.2013.00540)
Supplement: Figure S1 — Phosphopeptide motifs of extracted from peptides associated with proteins which have an absolute correlation coefficient greater than or equal to 0.8 with selected kinases based on the respective phosphorylation level profiles. The analysis was done for those kinases with at least 10 correlated proteins. Those proteins and their associated peptides were then interpreted as the kinase targets. motif-x (Schwartz and Gygi, 2005) was used with p-value threshold 0.01, the occurrence threshold was set to 10. [file DataSheet1.ZIP › supplementary table2.docx]

**Supplementary Table 2**. List of top 20 nodes with highest degree in the reconstructed network

| AGI | Degree | Function |
| --- | --- | --- |
| AT2G42320 | 28 | DNA.synthesis/chromatin structure |
| AT1G66200 | 23 | N-metabolism.ammonia metabolism.glutamine synthetase |
| AT2G18960 | 23 | transport.p- and v-ATPases.H+-exporting ATPase |
| AT5G47430 | 22 | RNA.regulation of transcription.putative transcription regulator |
| AT5G09850 | 22 | RNA.regulation of transcription.putative transcription regulator |
| AT2G27720 | 21 | protein.synthesis.ribosomal protein.eukaryotic.60S subunit.P2 |
| AT1G48590 | 21 | not assigned.no ontology.C2 domain-containing protein |
| AT1G76970 | 20 | cell.vesicle transport |
| AT3G11940 | 20 | protein.synthesis.ribosomal protein.eukaryotic.40S subunit.S5 |
| AT5G16880 | 20 | cell.vesicle transport |
| AT2G30710 | 20 | signalling.G-proteins |
| AT1G01050 | 19 | nucleotide metabolism.phosphotransfer and pyrophosphatases.misc |
| AT3G01310 | 19 | not assigned.unknown |
| AT1G53310 | 19 | Unknow |
| AT3G05420 | 18 | lipid metabolism.FA synthesis and FA elongation.acyl-CoA binding protein |
| AT3G44750 | 18 | RNA.regulation of transcription.HDA |
| AT4G30190 | 18 | transport.p- and v-ATPases.H+-exporting ATPase |
| AT5G61780 | 18 | RNA.regulation of transcription.Zn-finger(CCHC) |
| AT4G11270 | 17 | development.unspecified |
| AT3G55610 | 16 | amino acid metabolism.synthesis.glutamate family.proline.delta 1-pyrroline-5-carboxylate synthetase |
